# Addressing help-seeking, stigma and risk factors for suicidality in secondary schools: short-term and mid-term effects of the HEYLiFE suicide prevention program in a randomized controlled trial

**DOI:** 10.1186/s12889-023-17557-9

**Published:** 2024-01-08

**Authors:** Luna Grosselli, Susanne Knappe, Julia Baumgärtel, Ute Lewitzka, Jürgen Hoyer

**Affiliations:** 1 Fakultät Psychologie, Institut für Klinische Psychologie und Psychotherapie, Technische Universität Dresden, Dresden, Germany; 2 Werner-Felber-Institut e. V, Dresden, Germany; 3 Evangelische Hochschule Dresden (ehs), University of Applied Sciences for Social Work, Education and Nursing, Dresden, Germany; 4 Department of Psychiatry & Psychotherapy, Faculty of Medicine, Carl Gustav Carus University Hospital, Technische Universität Dresden, Dresden, Germany

**Keywords:** Suicide, Prevention, Program, School, Youth, Adolescents, Randomized-controlled trial

## Abstract

**Background:**

Suicidal ideation and suicide attempts present a serious public health concern among adolescents and young adults. School-based suicide prevention programs are a key tool for addressing this problem. However, more research is necessary to assess their effectiveness, acceptability, and safety. In response, the HEYLiFE suicide prevention program was developed to enhance help-seeking, reduce stigma towards suicidal peers and diminish risk factors for suicidality. This article presents the evaluation findings of the HEYLiFE program in German secondary schools.

**Methods:**

We conducted a randomized-controlled trial measuring short-term pre-post within-group effects in the intervention group only and mid-term effects at 6-months-follow-up compared to a waitlist-control group. Schools were assigned randomly to the intervention or control group (no blinding). We recruited students ≥12 years of age. Primary outcomes were knowledge about suicidality, attitudes towards suicidality, stigma towards a suicidal peer, help-seeking intentions and behaviours, risk factors for suicidality. The data was analysed with linear mixed models and generalized linear mixed models.

**Results:**

A total of *N* = 745 students participated (*n* = 353 intervention group, *n* = 392 control group). We observed favourable short-term effects on knowledge, attitudes towards suicidality and fear towards a suicidal peer. Unexpectedly, the program also led to an increase in desire for social distance and a decrease in prosocial emotions towards a suicidal peer. The mid-term effects of the program were exclusively favourable, resulting in enhanced attitudes towards help-seeking while protecting from a sharper rise in risk-factors for suicidality and from an increase in social distance. The program had more favourable effects on females and on students aged >13 years. The program was well-received by the students, and no serious adverse events were reported.

**Conclusions:**

These findings demonstrate the effectiveness of the HEYLiFE universal suicide prevention program in addressing variables associated with suicidal ideation and suicide attempts among adolescents on the mid-term. The short-term negative effects on stigma and more negative effects on males should be addressed in the future. Future evaluation studies should examine its effects on suicidality and its effectiveness within populations at high risk.

**Trial registration:**

The study was preregistered in the German Clinical Trials Register (registration number: DRKS00017045; registration date: 02/04/2019).

**Supplementary Information:**

The online version contains supplementary material available at 10.1186/s12889-023-17557-9.

## Background

Suicidality is a major topic of concern for adolescents’ mental health. The cumulative incidence at the age of 21 was estimated at 13.5% for any suicidal behaviour (12.7% for ideation, 6.6% for plans, and 4.0% for attempts) [1]. While suicidal thoughts and suicide attempts are rare before the age of 10, their incidence increases steeply after the age of 12 [2]. Adolescents with suicidal ideation face numerous barriers to accessing mental health services, with approximately 28% receiving any professional assistance [3]. Beyond structural barriers, individual factors like fear of hospitalization or stigmatization can also hinder help-seeking behaviours [3–5].

Suicidal behaviour is a complex phenomenon that is influenced by a multitude of factors, including mental disorders, adverse life events, challenging familiar situations, and personality traits [6]. Several theories explain the emergence of suicidal ideation and the transition from ideation to attempts (e.g., Interpersonal Theory of Suicide [7]; Integrated Motivational-Volitional model [8]). The cognitive and emotional risk factors for suicide attempts with the most robust empirical support include hopelessness, feeling of being disconnected from others (thwarted belongingness), feeling of being a burden for others (burdensomeness), and feeling defeated or trapped in a negative situation (entrapment) [9].

It is crucial to target these risk factors related to suicidality as early and systematically as possible for suicide prevention [10]. Fortunately, respective activities in schools have been developed over the past decade [11, 12]. Preventive measures include educational programs (e.g., Youth Aware of Mental Health, YAM) [13] screening programs for suicide risk (e.g., Columbia Suicide Screen) [14], gatekeeper education for school personnel, parents, and peers (e.g., Question Persuade and Refer, QPR) [15, 16], as well as postvention strategies [17]. In a large multinational randomized-controlled trial, the YAM program was effective in reducing suicide attempts and suicidal thoughts, while a gatekeeper training (QPR) and a screening program (ProfScreen) did not [13]. Recent meta-analyses have supported the effectivity of educational suicide prevention programs targeting risk and protective factors [12, 18].

Despite the evidence indicating the beneficial effects of educational suicide prevention programs, certain limitations have been raised. Not all implemented prevention programs in schools have proven effective [19] and some programs seem to have differential effects for specific groups (e.g., more favourable outcomes for females than for males) [20]. Also, there is substantial variation and uncertainty regarding their theoretical foundation [21]. Although reduced suicide attempts and suicidal ideation are the main outcomes of a suicide prevention program, these are rare phenomena and considerable sample sizes are required to detect changes [22]. Therefore, there has been a call to identify the most appropriate alternative suicide-related outcomes [23, 24]. Although adverse effects have been reported [25], evaluation studies rarely explicitly assess unwanted side effects (USE) and serious adverse events (SAE) [11] raising doubts about the safety of prevention programs. Ultimately, the implementation of evidence-based programs is hindered by significant practical barriers, such as low acceptability and high costs [12, 26]. Accessible and acceptable interventions with a solid empiric foundation are urgently needed and should be evaluated in high-quality trials with adequate outcomes considering both positive and negative effects [11, 22].

In response to these challenges, the HEYLiFE educational suicide prevention program for secondary schools was developed. A Delphi survey with leading suicide prevention experts was conducted beforehand to obviate the lack of coordination regarding the theoretical basis of prevention programs [10]. The HEYLiFE program was developed and evaluated according to these recommendations.

This paper describes the evaluation of the HEYLiFE prevention program in a longitudinal, randomized controlled trial (RCT). We hypothesized that participation in the program would lead to (1) an increase in mental health knowledge, (2) lower levels of maladaptive attitudes towards suicidality and stigma towards suicidal peers, (3) increased intentions for help-seeking and help-seeking behaviours, and (4) attenuation of risk factors for suicide ideation. We evaluated the efficacy both short-term (pre-post) and mid-term (6 months follow-up) and compared the results of an intervention group (IG) with changes in a waiting-control group (CG) at follow-up (parallel design, allocation ratio 1:1). Furthermore, we examined program acceptance and safety as a critical hallmark for later transfer into routine application in educational settings. Finally, we also explored program efficacy relative to gender and age group.

## Methods

### Study program and recruitment

The study was conducted as part of the activities of NeSuD [27], a research and prevention initiative aimed to increase awareness of suicidality and to promote access to sources of help (funding no. ZMVI1-2517FSB148, German Federal Ministry of Health). Since late 2017, the network has arranged meetings and workshops for local mental health care providers. Our team developed the HEYLiFE universal educational suicide prevention program for secondary schools (≥12 years) with support from the network.

To evaluate the HEYLiFE program, all secondary schools from the city of Dresden (Saxony, Germany; *n* = 165) were invited via email and telephone to participate in a randomised-controlled trial in April and May 2019. After primary school (4 years), students in Saxony can enrol in an academic secondary school (*Gymnasium*, 8 years), which prepares them for university, or in a general secondary school (*Oberschule*, 6 years), which prepares for vocational training in secondary education (*Berufsschule*, 2–3 years). While many students enrol in the *Berufsschule* directly after completing the *Oberschule*, there is no limit of age for the *Berufsschule*. We recruited schools of both the academic (Gymnasium) and vocational school types (Oberschule, Berufsschule). Schools were randomly assigned to either the IG or a waitlist CG with computer-generated random numbers a priori. Schools and participants were informed about the randomization process and the condition they were randomized to (no blinding). The schools were free to decide which classes would participate in the program. For the evaluation study, we included all students from the participating classes who were 12 years or older and had sufficient knowledge of the German language. We decided not to establish a maximum age limit to ensure that our sample would be representative of the German secondary school students' population.

### A priori sample size calculation

For our primary outcomes (knowledge, attitudes, stigma, risk-factors for suicidality and help-seeking intentions), small to medium effects through preventive interventions were reported in previous studies [28, 29]. Power calculations using simulations (1000 repetitions) of variance analyses with three time-points and two groups in R indicated that a total sample of *n* = 534 participants (baseline to follow-up) was needed to detect small effects (given α = .05, β = .80 and *ES* = .30) [30]. Considering possible design effects (cluster randomization) and a drop-out rate of 15% from T0 (baseline) to T1 (post-assessment) and 15% from T1 to T2 (follow-up-assessment), we aimed to recruit *N* = 744 students at baseline.

### Study sample

**Fig. 1:**
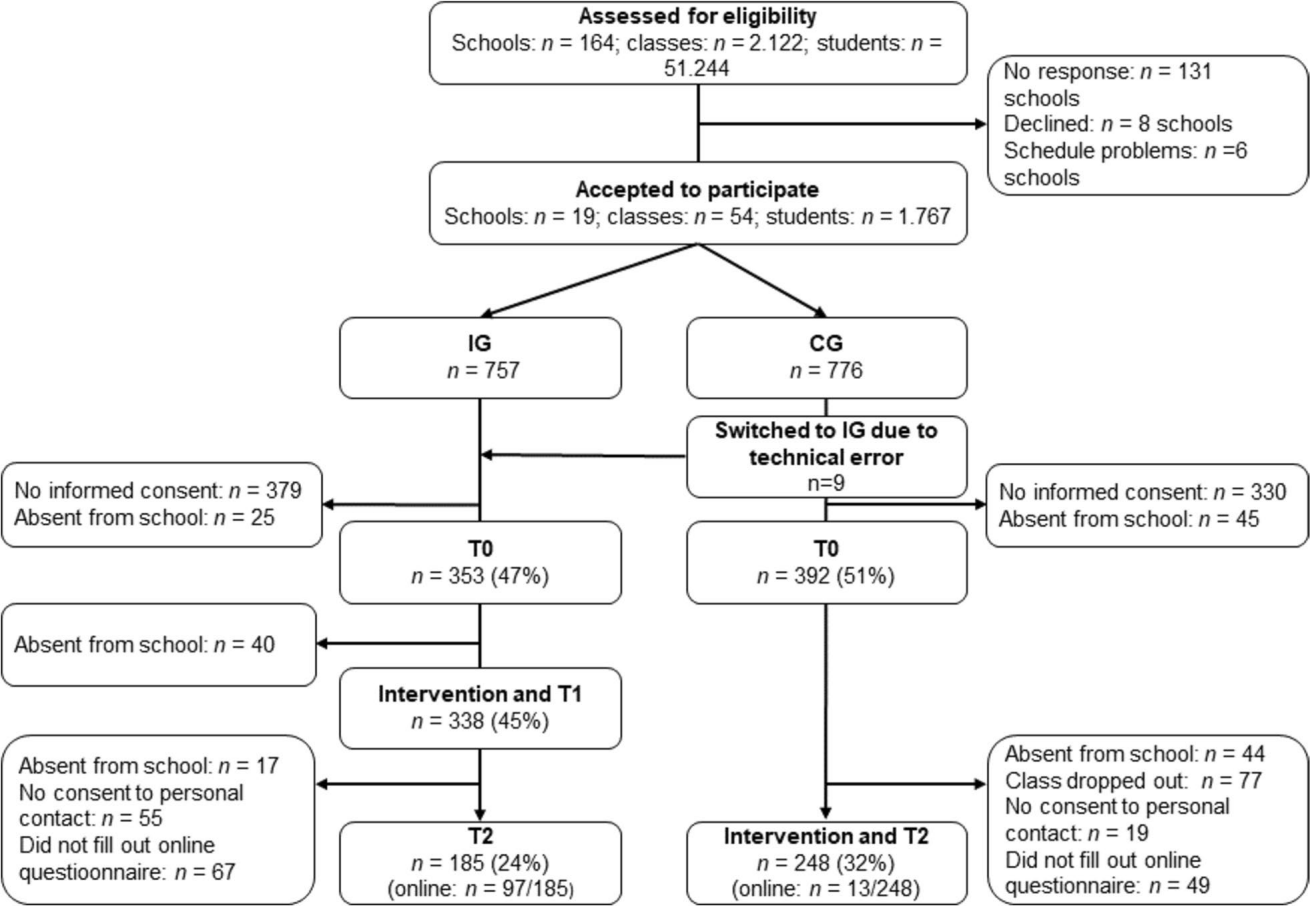
Flow Diagram of Participants and Drop-Out Reasons. Note. Technical error: Typing error in the invitation of the school (IG instead of CG)

Overall, 54 classes from 19 schools participated in the study. The first school enrolled in June 2019 and the last in September 2020 (last school out: 16.02.2021). The study ended as planned in March 2021. See Figure 1 for participation rates, loss-to-follow-up and drop-out reasons for schools, classes, and students.

At baseline, *n* = 353 and *n* = 392 students, respectively, were enrolled in the IG or CG. Gender proportions were almost balanced at baseline, with *n* = 308 (41.3%) males. Mean age was 15.5 years (*SD* = 2.3 years; *min* = 12, *max* = 42 years; *n* = 21 > 25 years old; Table 1). The IG and the CG had no significant difference regarding age, gender, migration background and mental health status. The CG, however, had a significant higher proportion of students in the academic school type. Given this imbalance, analyses were adjusted for school type.

**Table 1:** Demographic data and group differences at baseline (T0)

**Variable**	**Total (*N* = 745)**	**IG (*n* = 353)**	**CG (*n* = 392)**	**Comparison**
Age in years M (SD)	15.5 (2.3)	15.91 (3.9)	15.89 (3.4)	*T* = .08, *df* = 742, *p* = .93
12-13 years *n* (%)	205 (27.5)	103 (29.2)	102 (26.0)	*Χ*^2^ = 2.39, *df* = 3, *p* = .50
14-15 years *n* (%)	319 (42.8)	146 (41.4)	173 (44.1)	
16-17 years *n* (%)	104 (14.0)	53 (15.0)	51 (13.0)	
18+ years *n* (%)	116 (15.6)	50 (14.2)	66 (16.8)	
Gender				
Females *n* (%)	427 (57.3)	205 (58.1)	222 (56.6)	*Χ*^2^ = .91, *df* = 2, *p* = .64
Males *n* (%)	308 (41.3)	142 (40.2)	166 (42.3)	
Non-binary *n* (%)	10 (1.3)	6 (1.7)	4 (1.0)	
School type				
Academic/Gymnasium* n* (%)	248 (33.3)	89 (25.2)	159 (40.6)	***Χ***^**2**^** = 19.70,** ***df* = 1, *p* < .001**
Vocational *n* (%)	497 (66.7)	264 (74.8)	233 (59.4)	
Oberschule *n* (%)	356 (47.8)	211 (59.8)	145 (37.0)	
Berufsschule *n* (%)	141 (18.9)	53 (15)	88 (22.4)	
Migrant background *n* (%)	106 (14.2)	46 (13)	60 (15.3)	*Χ*^2^ = .73, *df* = 1, *p* = .39
Mental health status				
Depression (PHQ-9 => 11)* n* (%)	70 (9.4)	36 (10.2)	34 (8.7)	*Χ*^2^ = .33, *df* = 1, *p* = .57
Emotional/behavioural problems (SDQ)				
Abnormal *n* (%)	46 (6.2)	23 (6.5)	23 (5.9)	*Χ*^2^ = 2.74, *df* = 2, *p* = .25
Borderline *n* (%)	42 (5.6)	15 (4.2)	27 (6.9)	
Suicidal ideation (PSS Item 4)				
Last 2-weeks *n* (%)	26 (3.5)	16 (4.5)	10 (2.6)	*Χ*^2^ = 3.91, *df* = 2, *p* = .14
In the past *n* (%)	136 (18.5)	57 (16.1)	79 (20.2)	
Suicide attempts (PSS Item 5)				
Last 2-weeks *n* (%)	3 (0.4)	2 (0.6)	1 (0.3)	*Χ*^2^ = 1.48, *df* = 2, *p* = .48
In the past *n* (%)	54 (7.2)	22 (6.2)	32 (8.2)	

Bold prints indicate statistical significance at *p* < .05

*CG* Control Group, *IG* Intervention Group, *PHQ* Patient Health Questionnaire, *SDQ* Strengths and Difficulties Questionnaire, *PSS* Paykal’s Suicide Scale

It is important to note that the onset of the SARS-Cov-2-pandemic affected study participation (Figure 1). Because of the pandemic, four classes from the CG dropped out from the study before T2. Other schools found alternative solutions to allow participation despite school closures, reduction of class hours and contact limitations during the pandemic. The school personnel of 4 classes in the IG administered the T2 survey online during school hours. Further, we invited the students of 9 classes in the IG and 7 classes in the CG to participate singularly to our online-assessment from home. A portion of the study participants of those classes had previously agreed to personal contact with the study team (IG: *n* = 91/146; CG: *n* = 62/81). We invited those students per e-mail or per mail to fill in T2 questionnaires online from home. Three tablet computers, five e-watches and 20 vouchers worth 15 Euros were offered as an incentive.

There was no substantial group difference in the proportion of students that completed T0 assessments *after* the SARS-Cov-2-outbreak in Germany between IG (*n* = 87, 24.6%) and CG (*n* = 105, 28.0%). The proportion of completed T2 assessments was, however, clearly higher in the IG (*n* = 170, 91%; CG: n = 100, 40%). Moreover, measurement intervals between T0 and T2 assessment differed between the IG (*M* = 282 days, *SD* = 80) and in the CG (*M* = 160 days, *SD* = 51).

### Intervention

**Fig. 2:**
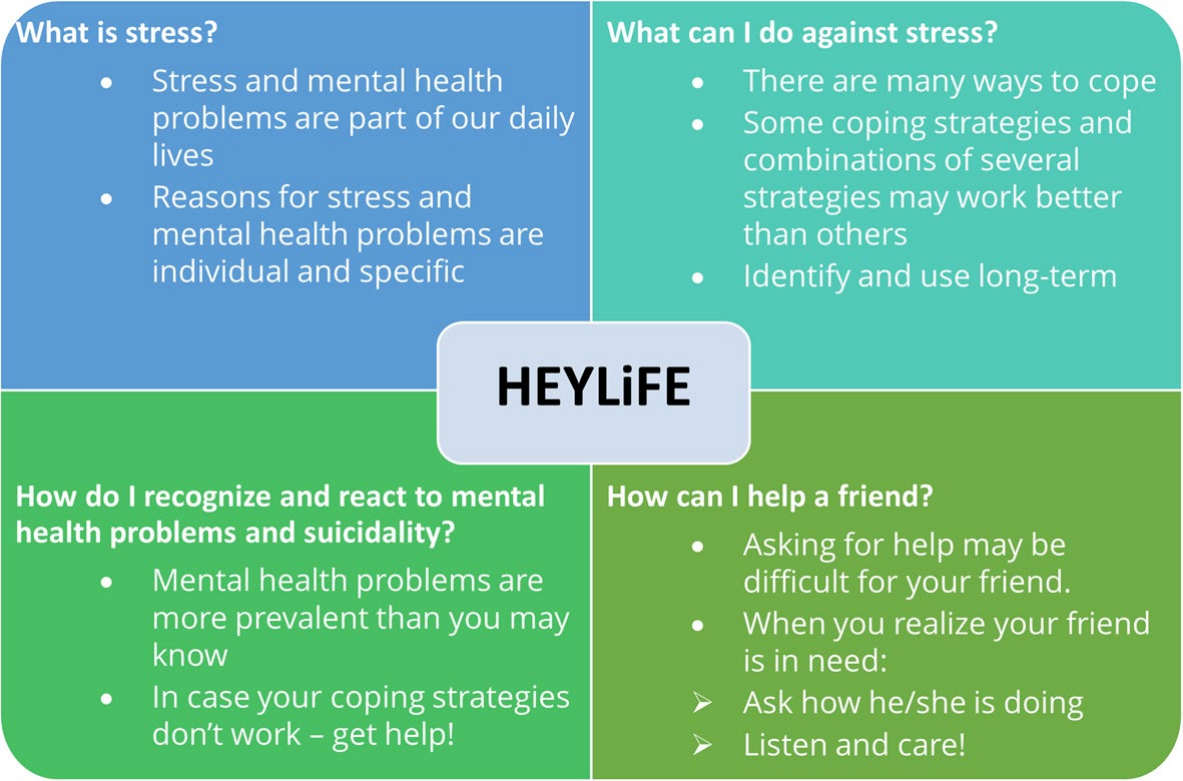
Contents of the HEYLiFE Prevention Program

HEYLiFE is a school-based suicide prevention intervention for students aged >12 years with a duration of 180 minutes deliverable in one or two sessions. It was developed by the first and second author, who have expertise in cognitive-behavioural therapy for children, adolescents, and adults. Forehand, we conducted an international three-stage Delphi survey with experts of suicide prevention [10] to gather suggestions about effective contents and methods in school-based suicide prevention. Following the suggestions of this survey, HEYLiFE was designed to reduce barriers to help-seeking, unfavourable attitudes towards suicidality and stigma towards suicidal peers. Furthermore, the program aims to promote mental health literacy, enhance coping strategies for difficult situations and mitigate individual risk factors for suicide attempts (feelings of hopelessness, isolation, burdensomeness, entrapment). The initial program was piloted in two classes of 14-year-olds. Students’ and teachers’ written and oral feedback was considered for revisions, resulting in an adaptation of wording and in the use of more interactive methods. Figure 2 depicts the contents of the final program. After an oral presentation on stress and mental health, students receive a booklet with information and are instructed to work individually and in small groups on exercises and discussions about stressors and coping mechanisms. Reading case vignettes of teenagers in their same age, they are asked to detect the signs of mental strain, depression, and suicidal behaviour, and to discuss potential means of offering assistance. The program also contains quizzes on myths and facts about suicidal behaviours and mental health care and provides information on local sources of help. Students finally engage in a role-play on how to ask for and offer support. The program was administered by master’s degree students in Clinical Psychology and related fields, who were trained in a one-day workshop. Overall, 13 trainers were involved. Teachers are not allowed in the room to allow students to open up; schools do not need to provide any technical support or personnel. HEYLiFE only uses few and inexpensive materials, namely a 5-page booklet, postcards on local health services for students, an instruction manual and re-usable case vignettes for program trainers. Although additional costs for the 1-day training of new trainers may originate in future, we wish to keep the program costs at a minimum in the future to assure the accessibility and facilitate implementation.

### Program safety

To ensure program safety, a masked procedure was used to identify students who reported suicidal behaviour in the past two weeks. At the beginning of the assessment, students were informed that some of them would be selected randomly to provide feedback after program administration. When items on severe suicidal ideation, or suicide attempts (Paykel’s Suicide Scale, PSS; Items 4 or 5) [31] were positively affirmed (*n* = 46 students), trainers registered the students’ code (“index students”). In addition, the code of the same number of non-suicidal students was listed. Students with the selected codes were then asked to enter separate rooms for the feedback interview. While index students were evaluated by at least one trainer to determine their suicide risk, non-suicidal students were interviewed about satisfaction with the program. When acute suicidality was given, further steps were taken (informing legal guardians, school social workers or therapists).

### Assessment procedures

Administration of the intervention was accompanied by assessments at T0 (1 week before intervention), T1 (immediately after intervention) and T2 (6-month-follow-up-assessments) in the IG. The CG completed the assessments at T0 and T2. This means that short-term effects are assessed in a pre-post design without CG, while we can compare both groups for the mid-term effects. Knowledge, attitudes, stigma, help-seeking intentions, and suicidality were measured at T0, T1 and T2. Demographics, help-seeking behaviours, risk-factors for suicidality, depression and mental health problems were only measured at T0 and T2 since no note-worth changes were expected between T0 and T1. Data collection was predominantly based on paper-pencil assessments in class; due to the SARS-Cov-2-pandemic T2 assessments were occasionally administered online via the SoSci platform [32].

Assessments on knowledge, attitudes towards suicidality and the case-vignettes used for stigma assessments were available only in English and were thus translated in two steps. First, two people with German mother tongue and excellent English knowledge translated the English items into German. Then, a bilingual person compared both versions and opted for the most adequate translation. The vignettes were slightly adapted to fit the German culture.

### Primary outcomes

*Knowledge on suicidality* was assessed by the Signs-of-Suicide-knowledge-questionnaire (SOS-k) [33]. The SOS-k consists of 7 dichotomous items (true or false). The 3-month test-retest reliability was low in a group of middle-school-students (*r* = .33) [34]. For our study, items 1 to 6 were used since item 7 (alcohol consumption and suicidality) was beyond the focus of our intervention. The six-moths test-retest reliability in the CG in our study was *r* = .50.

*Attitudes towards suicidality* were measured using the attitudes scale of the SOS questionnaire (SOS-a) [33] with 10 Likert-Scale items ranging from 1 “not at all” to 5 “definitely”. Higher values indicate more adaptive attitudes (e.g., “If a friend told me he/she is thinking about committing suicide, I would tell it to an adult at school”). The authors reported acceptable internal consistency (α = .73) [34]. The internal consistency in our sample was low (α = .56).

#### Stigma

Emotional reactions and social distance towards a suicidal peer were assessed using a case vignette depicting an adolescent with depression and suicidal thoughts [35]. The Emotional Reactions towards the Mentally Ill Scale (ERMIS) [36] presents 12 adjectives indicating emotional reactions to the peer in the vignette, rated from 0 “not at all” to 4 “extremely”. As suggested by the authors, we ran a confirmative factor analysis and used the factor scores for further analysis. This scale was found to have a good construct validity [36]. Reproducing the structure of the original article, we obtained the three latent factors “rejection”, “fear” and “prosocial reaction”. The *CFI* of the model was satisfactory (*CFI* = .93). The internal consistency of the scales was acceptable (α = .65, α = .64, α = .63). Furthermore, we assessed social distance with the 5-item social distance scale (SDS) [37, 38]. Participants rated how likely they are willing to engage in a social activity with the depressive peer in the vignette from 0 “not at all likely” to 4 “yes, definitively”. Higher sum scores indicate higher levels of willingness to engage (i.e., less social distance). The scale demonstrated good construct reliability, internal consistency (α = .89) and discriminant validity for the use with adolescents [39]. Internal consistency was good (α = .87) in this study.

#### Help-seeking intentions and behaviour

We used the General Help-Seeking Questionnaire (GHSQ) [40] to assess how likely someone would seek help for a mental health problem from different help sources on a scale from 1 “very unlikely” to 7 “very likely” (*help-seeking intention*). The authors report a sufficient internal consistency (α = .70) and 3-weeks test-retest-reliability (*r* = .86) for help-seeking intention for “emotional and mental health problems”. The authors encourage users to adapt the items regarding the specific problem and help sources. Likewise, we asked for the intention to seek help for an emotional or mental health problem. Since we aimed to encourage participants to specifically seek professional help in case of suicidality, we only considered professional help sources (psychologist, doctor, social worker, e-mail counselling service, telephone counselling service) in the analyses and used the highest score among these items to determine the intention to seek professional help [41]. The Actual Help-Seeking Questionnaire (AHSQ) [42] was used to assess *help-seeking behaviours*. The AHSQ contains the same items as the GHSQ, and participants indicate whether they did (“yes”; 1 point) or did not (“no”; 0 points) reach out for help for emotional or mental health problems to each source of help in the last 6 months. For analyses, we considered whether any professional help resource was sought (1-yes, 0-no).

#### Risk factors for suicidality

We developed a 4-item Risk Factor Scale (RFS; Additional file 1) based on items from the BeMind-study [43]. The items represented feelings of burdensomeness, thwarted belongingness, hopelessness, and entrapment over the last two weeks on a scale between 0 “not at all” and 3 “nearly every day”. We selected these items since they depict central concepts of widely accepted theories on the development of suicidal ideation and suicide attempts [7–9]. A sum score across all items was calculated, higher scores indicating higher risk for suicidality in the past two weeks. Good internal consistency at α = .81, as well as positive moderate correlations with depression (Patient Health Questionnaire, PHQ-9; *r* = .66; *p*< .001) [44] and suicidality (PSS suicidal ideation, *r* = .30, *p* < .001; suicide attempts, *r* =.23, *r* < .001) indicating good convergent validity were observed in our study.

### Secondary outcomes

*Mental health status* was measured using the PHQ-9 for depression in the past two weeks [44, 45] and the Strength and Difficulties Questionnaire to screen for emotional and behavioural problems (SDQ) [46, 47]*.* Suicidality was assessed using the five-item PSS [30]. The scale was proved to have good internal consistency (*ω* = .82) and acceptable test-retest reliability with adolescents (*r* = .61) [48]. For this study, participants indicated presence of suicidality “in the past 2 weeks”, “any time before” or “never”. Lifetime suicidality was defined as suicidality in the past 2 weeks or before. The internal consistency for the whole scale in our study was *ω* = .85 (in the past two weeks) and *ω* = .80 (lifetime). We used item 4 (severe suicidal ideation) and item 5 (suicide attempts) as indicators for severe suicidality [13].

#### Acceptability

A 7-item scale (range: 1 “not at all” to 5 “very”) to evaluate program acceptability was developed based on the theoretical framework by Sekhon, Cartwright and Francis [49]. The scale demonstrated good internal consistency in this study (α = .79).

#### Unwanted side effects and serious adverse events

Concordant with clinical studies, trainers documented USE and SAE observed by the trainers or by school personnel during and immediately after assessment procedures (Additional file 2).

### Statistical analyses

IBM SPSS Statistics 27 [50] and R (lavaan, lme4) [51, 52] were utilized for statistical analyses. Hypotheses were tested two-sided using linear mixed models (LMM) for ordinal outcomes and generalized linear mixed models (GLMM) for binary outcomes. For short-term effects, we compared the score of the IG before and after the intervention (fixed effect of time). For mid-term effects (6 to 9 months follow-up), we compared changes in the IG with changes in the CG (fixed effect of the interaction group*time). To achieve better interpretation, we computed separate models for each group when the interaction group*time was significant. We controlled for school type. In further exploratory analyses, we added gender/age group (12-13 y., 14-16 y., 17+ y.) and the interaction between gender*time/age group*time(short-term) or between gender*time*group/age group*time*group (mid-term) as fixed effects. In a second step, we ran all analyses for each gender and age group separately. The data of students was nested in school-classes to control for random effects at student and class-level. A-priori alpha level was set at *p* < 0.05. Reporting an effect size for the difference between groups as Cohen’s *d* would not have been appropriate for our data, because most of our outcome variables were not normally distributed. Instead, we determined the odds ratio (*OR*) of the IG achieving favourable values for each variable, comparing the IG to both to its own results at T0 (short-term) and to the results of the CG (mid-term). For ordinary variables, a participant was considered to reach a favourable value if their (rounded) score could be interpreted as “rather good” or “very good” on the Likert-scale it referred to (e.g., a score ≥ 3.5 on a 1-5 Likert-scale). No adjustments were made for multiple testing because the individual tests were related to individual hypotheses and adjustment would have treated them as a global hypothesis [53].

To explore intervention efficacy by pandemic onset, we compared mental health indicators (depression, emotional or behavioural problems, suicidality, risk-factors for suicidality) between participants with T2 assessments before (*n* = 164) and after (*n* = 270) the onset of the pandemic in Germany (first known Corona case in Germany on 28^th^ January 2020). We conducted the analysis using Chi-squared-test, t-test or Mann-Whitney-U-Tests according to requirements for level of measurement, homoscedasticity or normal distribution.

## Results

### Primary outcomes

See Table 2 for descriptive statistics at T0. The results of the GLM and GLLM for the primary outcomes are shown in Table 3.

**Table 2:** Overall group and time differences in primary outcomes

	**T0**		**T1**		**T2**	
**Variables and Group**	*M* (*SD*)	*N*	*M* (*SD*)	*N*	*M* (*SD*)	N
Mental Health Knowledge						
Knowledge (SOS-k)						
IG	3.80 (1.23)	339	4.57 (1.28)***	327	4.17 (1.24)	175
CG	3.92 (1.23)	377	/	/	4.09 (1.23)	246
Attitudes and Stigma						
Attitudes Towards Suicide (SOS-a)						
IG	3.36 (.58)	335	3.45 (.55)**	319	3.41 (.61)	176
CG	3.43 (.52)	362	/	/	3.37 (.55)	245
Stigma Rejection (ERMIS)^a^						
IG	.01 (.40		.05 (.45)	335	-.01 (.33)	181
CG	-.04 (.26)	391	/	/	-.02 (.26)	248
IG	.07 (.94)		-.21 (.93)***	335	-.12 (.97)	181
CG	.16 (.90)	391	/	/	.04 (.97)	248
Stigma Prosocial Reactions (ERMIS)^a^						
IG	.03 (.74)		-.15 (.83)***	335	-.10 (.80)	181
CG	.18 (.61)	391	/	/	-0.3 (.68)	248
Stigma Willingness for Social Contact (SDS)						
Intervention	8.33 (3.84)	344	7.84 (4.10)*	325	**7.94 (4.17)**	181
Control	8.89 (3.50)	378	/	/	7.87 (3.85)***	243
Risk Factor Scale (RFS)						
IG	.30 (1.11)	331	/	/	2.15 (2.71)***	172
CG	.23 (.85)	357	/	/	3.48 (3.26)***	243
Help-Seeking						
Intention to Seek Professional Help (GHSQ)						
IG	4.25 (1.86)	345	4.00 (1.91)	331	**4.44 (1.86)**	176
CG	4.47 (1.78)	383	/	/	4.21 (1.82)*	247
Help-Seeking Behaviour (AHSQ)	*n* (%)	*N*			*n* (%)	*N*
IG	61 (14.9)	340	/	/	27 (6.6)	172
CG	82 (19.8)	379	/	/	45 (10.8)	241

Significant effects of the intervention (main effect of time at T1; interaction between time*group at T2) at p<.05 are highlighted in bold

*CG* Control Group, *IG* Intervention Group, *SOS-k* Signs of Suicide Questionnaire, knowledge scale, *SOS-a* Signs of Suicide Questionnaire, attitudes scale, *ERMIS* Emotional Reaction to the Mental Ill Scale, *SDS* Social Distance Scale, *RFS* Risk Factors Scale, *GHSQ* General Help Seeking Questionnaire, *AHSQ* Actual Help Seeking Questionnaire

^a^weighted sum score (weight: CFA loading on latent factor)

^*^p<.05 **p>.01 *** *p*<.001 (significant effect of time/change from T0 for each group)

**Table 3:** Short- (T1) and Mid-term Effects (T2) of the Intervention

	**T1**				**T2**			
**Variables and Group**	**Estimate**	***F***	***p***	***CI* (95%)**	**Estimate**	***F***	***p***	***CI (95%)***
Mental Health Knowledge								
Knowledge (SOS-k)								
Time^a^	**.84**	131.34***	**<.001**	**.70, .98**	.19	17.42***	<.001	.01, .37
School Type^b^	-.51	4.08	.06	-1.04, .02	-.25	3.42	.07	-.51, .02
Group^c^	/	/	/	/	.08	.02	.90	-.23, .40
Time*Group	/	/	/	/	.20	2.05	.15	.07; .46
Attitudes and Stigma								
Attitudes Towards Suicide (SOS-a)								
Time^a^	**.08**	6.55*	**.01**	**.02, .14**	-.03	.13	.71	-.11, .03
School type^b^	.13	1.85	.19	-.07, .33	.07	1.67	.20	-.04, .19
Group^c^	/	/	/	/	.02	.25	.61	-.12, .15
Time*Group	/	/	/	/	.09	2.45	.12	-.02, .20
Stigma Rejection (ERMIS)								
Time^a^	.03	1.47	.23	-.02, .09	.02	.04	.85	-.02, .07
School type^b^	.10	2.06	.17	-.05, .26	.07	7.74**	.009	.02, .11
Group^c^	/	/	/	/	-.002	.46	.50	-.07, .06
Time*Group	/	/	/	/	-.04	1.22	.27	-.10, .03
Stigma Fear (ERMIS)								
Time^a^	**-.29**	38.29***	**<.001**	**-.39, -.20**	-.12	12.89***	<.001	-.01, -.24
School type^b^	-.16	.88	.36	-.53, .20	-.08	.73	.40	-.27, .11
Group^c^	/	/	/	/	-.16	1.88	.18	-.38, .05
Time*Group	/	/	/	/	-.08	.71	.40	-.25, .10
Stigma Prosocial Reactions (ERMIS)								
Time^a^	***-.17***	17.41***	***<.001***	***'-.25, '-.09***	-.19	22.44***	<.001	-.16, .09
School type^b^	-.20	2.02	.17	-.51, .10	-.07	1.11	.30	-.21, .07
Group^c^	/	/	/	/	-.08	2.85	.10	-.24, .08
Time*Group	/	/	/	/	.06	.88	.35	-.07, .19
Stigma Willingness for Social Contact (SDS)								
Time^a^	***-.46***	5.93*	***.02***	***-.84, -.09***	-1.05	16.09***	<.001	-1.51, -.60
School type^b^	-.55	.68	.42	-1.97, .86	-.19	.26	.61	-.93, .55
Group^c^	/	/	/	/	.12	.44	.51	-.72, .97
Time*Group	/	/	/	/	**.71**	4.32*	**.03**	**.04, 1.40**
Help-Seeking								
Intention to Seek Professional Help (GHSQ)								
Time^a^	-.18	2.59	.11	-.39, .04	-.26	.01	.92	-.51, -.003
School type^b^	-.14	.30	.59	-.69, .41	.09	.44	.51	-.19, .37
Group^c^	/	/	/	/	.15	.54	.47	-.22, .51
Time*Group	/	/	/	/	**.49**	6.38*	**.01**	**.11, .88**
Help-Seeking Behavior (professional help; AHSQ)								
Time^a^	/	/	/	/	.48	8.53**	.004**	-.04, .98
School type^b^	/	/	/	/	-.72	4.45*	.04*	-1.40, -.05
Group^c^	/	/	/	/	.48	1.59	.21	-.33, 1.30
Time*Group	/	/	/	/	-.11	.10	.75	-.59, .82
Risk Factor Scale (RFS)								
Time^a^	/	/	/	/	3.39	442.52***	<.001	3.06, 3.73
School type^b^	/	/	/	/	-.06	.05	.82	-.59, .47
Group^c^	/	/	/	/	-1.36	6.12*	.02	-1.97, -.74
Time*Group	/	/	/	/	**-1.44**	31.85***	**<.001**	**-1.94, -.94**

Results of LMM and GLMM; bold prints indicate significant effects of the intervention at *p* <.05 in the desired direction (main effect of time for T1, interaction time*group for T2); ***bold italics are significant effects in an unwanted direction***;* CG* Control Group, *IG* Intervention Group, *SOS-k* Signs of Suicide Questionnaire, knowledge scale, *SOS-a* Signs of Suicide Questionnaire, attitudes scale, *ERMIS* Emotional Reaction to the Mental Ill Scale, *SDS* Social Distance Scale, *RFS* Risk Factors Scale, *GHSQ* General Help Seeking Questionnaire, *AHSQ* Actual Help Seeking Questionnaire. For effects by gender and age group, see additional material

^a^Reference category for the estimates: T1 or T2

^b^Reference category for the estimates: Professional school branch

^c^Reference category for the estimates: IG

^*^p<.05, **p<.01, ***p<.001

### Knowledge on suicidality

At T0, knowledge on suicidality in the total sample was moderate (SOS-k; *M* = 3.86, *SD* = 1.23; scale: 0-6), with only 31.6% of the students reaching a high level of knowledge (≥ 5 out of 6 points). Analyses revealed a significant increase in knowledge at T1 in the IG (SOS-k; Table 3). The odds ratio (OR) for attaining a high level of knowledge after the training as compared to before was *OR* = 3.57 (*p* < .001; 95% *CI* [2.58, 4.93]). At T2, there was a significant main effect of time (*p* < .001), but no significant interaction between time and group (*p* = .15). In comparison to a student in the CG, a student belonging to the IG exhibited an *OR* = 1.11 (95% *CI* [.75, 1.64]) of achieving a high level of knowledge at the follow-up stage.

### Attitudes towards suicidality

Students’ attitudes towards suicidality were mixed at baseline (SOS-a; *M* = 3.39, *SD* = .55; *M* range for SOS-a items = 2.24 – 4.66). Since the internal consistency of the scale was low, we also computed the analysis at the item level (Additional file 3).

There was a small, but significant increase in total adaptive attitudes towards suicidality at T1 in the IG (*p* = .01; Table 3), with an *OR* = 1.10 (95% *CI* [.81, 1.50]) to have overall more adaptive attitudes after the program compared to before. At the item level, significant changes were reached for the reversed items “[If a friend told me they were feeling suicidal…] I would not know what to do” (*p* < .001; *OR* = 1.88, 95% *CI* [.1.38, 2.58]) and “…I would keep it a secret” (*p* < .001; *OR* = 1.32, 95% *CI* [.96, 1.80]; Additional file 3).

At T2, there was no significant main effect or interaction of time and group (interaction: *p* =.12; *OR* = 1.00, 95% *CI* [.68, 1.47]). At the item level, a significant time*group interaction in the hypothesized direction was found only for the reversed item: “If someone wants to take their own life, there is no much I can do“ (*p* = .04; *OR* = 1.18, 95% *CI* [.78, 1.78]; Additional file 3).

### Stigma towards a suicidal peer

At T0, students displayed a low to medium stigma level regarding emotional reactions towards a suicidal peer (ERMIS). Specifically, they showed low levels of rejection (91.5% of students with a low rejection level), as well as medium levels of fear (42.6% with a low fear level) and prosocial emotions (45% with a high level of prosocial emotions). For the IG, there was no significant main effect of time at T1 (*p* < .23; low levels of rejection: *OR* = .76, 95% *CI* [.46, 1.24]). The students of the IG reported significantly less fear after intervention (*p* < .001; low levels of fear: *OR* = 1.73, 95% *CI* [1.28, 2.34]). Unexpectedly, there was a significant main effect of time with a *decrease* on the prosocial emotions scale (*p* < .001; high levels of prosocial emotions; *OR* =.77, 95% *CI* [.56, 1.06]). At T2, the interaction time*group was not significant for all three scales (anger: *p* = .27; *OR* = 1.36, 95% *CI* [.63, 2.90]; fear: *p* = .40; *OR* = 1.26, 95% *CI* [.85, 1.84]; prosocial emotions: *p* = .35; *OR* = .93, 95% *CI* [.61, 1.42]; Table 3), while there was a significant, negative main effect of time on fear (*p* < .001) and prosocial emotions (*p* < .001).

The desire for social distance (SDS) was at a moderate level before the intervention, with 42.5% of the students showing low levels on this variable. A short-term effect in the IG was observed at T1, showing a *decrease* in willingness to interact with a peer with suicidal thoughts (*p* = .02; *OR* = .92, 95% *CI* [.68, 1.26]; Table 3). On the other hand, in addition to a significant, negative main effect of time (*p* < .001) there was a significant group*time interaction at T2 (*p* = .03). A significant decrease in willingness to interact with a suicidal peer was found only in the CG (*p* = .03; *OR* =1.17, 95% *CI* [.79, 1.75]; Table 2; Additional file 4).

### Help-seeking

At baseline, 48.6% of students would rather get professional help if experiencing mental or emotional problems (GHSQ), while 19.9% had received professional help in the past 6 months (AHSQ). The main effect of time on help-seeking intention was not significant at T1 in the IG (*p* = .11; *OR* = .93, 95% *CI* [.69, 1.26]; Table 3). The interaction between time*group was significant at T2 (*p* = .01; Table 3), with a significant decrease in help-seeking intention in the CG only (Table 2; Additional file 4). The *OR* for a student to have high seeking intentions at T2 was 1.53 for the IG compared to the CG (95% *CI* [1.04, 2.26]). The time*group interaction had no significant effects in terms of the number of students receiving actual professional help at T2 (*p* = .75; *OR* = .81, 95% *CI* [.48, 1.36]; Table 3).

### Risk factors for suicidality

Please note that the risk factors were assessed at T0 and T2 only. At T0, 89.9% stated that they did not experience any of the suicide-related mental states described in the past 2 weeks (RFQ). At T2, we found a significant effect of time and a time*group interaction (*p* < .001; Table 3). While both IG and CG showed a significant increase in risk factors over time, the increase in the IG was significantly less pronounced (Table 2; Additional file 4). The *OR* for not experiencing any of the described suicide-related mental states was 1.87 for students in the IG compared to students in the CG at T2 (95% *CI* [1.23, 2.85]).

### Secondary outcomes

#### Program acceptability and safety

Students indicated high acceptability of the prevention program (*M* = 3.97, *SD*=.66; range 1 to 5). Based on safety procedures, *n* = 46 students (*n* = 22 IG, *n* = 24 CG) were evaluated after reporting acute suicidality. None of these interviews revealed any relationship between program participation and suicidality. Regarding USE, negative emotional reactions were observed twice during or immediate to the program (crying, leaving the room for some time). According to participants, their reactions have been related to emotional problems or bereavement or suicide in the family. Across the study period, no SAE was observed or has been reported to the study team. Table 4 depicts the number of students who reported suicidal ideation and suicide attempts in the last 2 weeks or lifetime at T0, T1 or T2. Of note, one further suicide attempt of a student who did not participate in T2 has been reported to the study team between T1 and T2 in the IG. No cases of suicide during the study period are known to the study team. There were no significant mid-term effects on acute or life-time severe suicidal ideation or suicide attempts (Additional file 5).

**Table 4:** Suicidal ideation and suicide attempts (in the past two weeks and lifetime)

	**T0 (Baseline)**		**T1 (Post)**		**T2 (Follow-up)**	
	***n* (%)**	***N***	***n* (%)**	***N***	***n* (%)**	***N***
**Suicidal Ideation Past 2 Weeks (PSS Item 4)**						
Intervention	16 (4.6)	346	18 (5.5)	329	9 (5.1)	175
Control	10 (2.6)	379	/	/	13 (5.3)	243
**Suicide Attempts Past 2 Weeks (PSS Item 5)**						
Intervention	2 (.6)	348	4 (1.2)	329	2 (1.1)	176
Control	1 (.2)	383	/	/	2 (.8)	242
**Suicidal Ideation Lifetime (PSS Item 4)**						
Intervention	73 (21.1)	346	58 (17.6)	329	29 (16.6)	175
Control	88 (23.2)	379	/	/	52 (21.4)	243
**Suicide Attempts Lifetime (PSS Item 5)**						
Intervention	24 (6.9)	348	32 (9.7)	329	11 (6.3)	176
Control	32 (8.4)	383	/	/	15 (6.2)	242

#### Effects of the pandemic onset on mental health

Suicidal ideation (PSS Item 4; *χ*^*2*^ =.37, *p* = .54) and suicide attempts (PSS Item 5; *χ*^*2*^ = .64, *p* = .42) as well as emotional and behavioural symptoms (SDQ; *t* = -.24, *p* = .81) did not differ between students completing T2 before and after the onset of the Sars-Cov2-pandemic in Germany. However, students completing T2 after the onset of the pandemic reported higher levels of depression (PHQ-9; *U* = 33259.5, *p* < .001) and a higher risk factor score for suicidality (RFS, *U* = 24806.0, *p* < .001) than students completing it before.

#### Exploring intervention efficacy by gender and age group

The complete results of GLMs and GLLMs with gender as a further predictor are shown in the Additional file 6. Gender had a main effect on rejection, fear, and prosocial emotions (ERMIS; *p* < .001), with males showing more stigmatizing emotional reactions than females. A significant time*gender interaction was found for rejection (*p* = .004) and prosocial emotions (*p* < .001) whereby only males showed a significant increase in rejection (males: *p* = .02; females: *p* = .21) and decrease in prosocial emotions (males: *p* > .001; females: *p* = .06) from T0 to T1.At T2, gender had a significant main effect on rejection, fear, and prosocial reactions (ERMIS; *p* < .001), with males showing more stigmatizing emotional reactions than females. A significant effect of the interaction time*group*gender was found for prosocial reactions (*p* = .01), social distance (*p* = .04) and help-seeking behaviour (*p* < .001) with more favourable results for females than for males of the IG (Additional files 7, 8).

As for age group, in the short-term analyses age group had a significant main effect (*p* < .05) on all variables except for rejection, with higher results for older students. The interaction time*age group was only significant for prosocial reactions (ERMIS; *p* = .04). The increase in knowledge from T0 to T1 was significant for all age-groups (SOS-k; *p* < .001). Only the age group 17+ had a significant improvement in attitudes towards help-seeking (*p* = .01) and prosocial reactions (*p* <.001). Time had a negative effect on social distance (*p* = .004) and help-seeking (*p* = .05) intention in 12-13-year-olds. As for mid-term effects (T0-T2), age group had a significant fixed effect (*p* < .05) on all outcomes except fear and rejection, with higher values for older students. The interaction age group*time*group was significant for knowledge (SOS-k; *p* = .01) and risk-factors (RFQ; *p* < .001). In the 12-13 years olds, prosocial reaction increased in the IG, but decreased in the CG (*p* = .05). Furthermore, there was an increase in knowledge only for the youngest group in the IG compared to the CG (*p* < .01). In contrast, in the age-group 17+, the CG had a higher increase in knowledge than the IG (*p* = .02). Also, the CG only had higher risk factors for suicidality (RFQ) than the IG for the age groups 14-16 (*p* < .001) and 17+ (*p* = .004).

## Discussion

The prevention of suicidality is a fundamental topic in the field of adolescent mental health [12]. Given the significant amount of time adolescents spend in school, it is an obvious setting for implementing suicide prevention strategies. The present school-based RCT examined the effects of the HEYLiFE suicide prevention program on a sample of *n* = 745 German secondary school students (≥ 12 years). Effects were assessed immediately following the intervention (short-term) and compared to a waiting-control group at follow-up (6-9 months later, mid-term). Our analyses revealed favourable short-term effects, including an enhancement in suicide knowledge and adaptive attitudes towards suicidality and a decrease in fear towards a suicidal peer. Prosocial emotions and willingness to interact with a suicidal peer, however, were reduced immediately after the intervention. No effects were observed for rejection towards a suicidal peer and help-seeking intention. Despite we observed an increase in knowledge and a decrease in fear and prosocial emotions at follow-up, this effect was not limited to the IG and thus not attributable to our intervention. Nonetheless, the program had a favourable effect on help-seeking intentions, risk-factors for suicidality and social distance at follow-up. No effects at follow-up were found for attitudes towards suicidality, rejection and prosocial emotions towards a suicidal peer and help-seeking behaviours. Interestingly, the program seemed to have a less favourable impact on males than on females for rejection and prosocial reaction in the short-term, and on social distance and help-seeking behaviours in the mid-term. Also, favourable effects on suicide risk factors were only observed in participants aged 14 years or older. The HEYLiFE intervention was rated positively by participants. No SAEs related to the intervention were documented.

Overall, the HEYLiFE prevention programs showed positive effects on the suicide related variables risk factors for suicide and help-seeking in the mid-term. These results support recent reviews and meta-analyses that suggest that curriculum-based prevention programs in schools are a valuable tool for suicide prevention among adolescents [11, 12, 18]. While our sample was insufficient to examine direct effects on suicidality, our study showed a favourable, mid-term impact on mental states directly related to suicide attempts, such as burdensomeness and entrapment. This suggests that HEYLiFE successfully improves skills to deal with stress and crises, and therefore could prevent the transition between suicidal ideation and suicide attempts [9]. Furthermore, the program seemed to reduce internal barriers for help-seeking, which could contribute to protecting young people at risk. The significance of this outcome is heightened by its alignment with expert recommendations for suicide prevention programs [10].

However, we also observed mixed effects immediately after the intervention. Unwanted side effects after suicide prevention programs have also been documented in earlier studies; these effects were particularly evident among males, racial minorities, and students at risk of suicide [23, 54, 55]. In our case, students (especially males and 12–13-year-olds) showed greater emotional distance from a suicidal peer immediately after the intervention compared to T0. Since we did not have a control group at T1, and we found more emotional distance in both groups at T2, this could also be an effect of weariness due to the repetition of the assessments. The set of questionnaires was rather time-consuming, and students complained that it was difficult to relate to the hypothetical person described in the vignettes. The inclusion of a control group and the use of alternative instruments could ensure that the intervention does not cause discomfort towards suicidal peers. Importantly, any unwanted negative effect was short-lived; at follow-up, only positive effects were documented.

Further potential for improvement was found regarding the effects on knowledge and attitudes since these did not last over time. Longer programs or a repetition of the contents over time could be necessary to achieve more durable changes. Similar to previous studies [20], males appeared to benefit less from the intervention. These results reassert the importance of crafting tailored interventions for male students [20]. While the 12-13 age group did have mid-term benefits for knowledge and pro-social reactions, it was the only age group with negative short time effects and the only group where the intervention did not have protective effects for risk-factors. For this reason, the HEYLiFE intervention seems more promising for students >13 years of age. In addition, no changes in actual help-seeking behaviour towards professional help resources were observed. This may be due to the low base rate of such events (as the analyses were conducted in a general population sample with a limited need for professional help) or an insufficient sample size or time-interval [22]. Likewise, it is possible that changing help-seeking *intentions* is not sufficient to promote actual help-seeking activities in young people. Interventions targeting systemic barriers (such as difficult accessibility and low availability of help) seem to be important here. Consequently, future studies should thus investigate the effects of HEYLiFE on a larger time-interval, assess pathways to help, and to examine effects on populations at risk.

This evaluation study significantly adds to the knowledge about school-based suicide prevention, particularly as RCTs as the one presented are still rare [56]. To our knowledge, the HEYLiFE prevention program is the first, which followed expert suggestions for the selection of contents and methods [26] since no accepted theoretical base for such programs existed up to date [21]. Of note, there is still limited empirical evidence regarding what exactly works in curriculum-based suicide prevention [12], thus expert knowledge is a very good starting point to generate programs that reflect the state of the art [57]. Program development was further informed by feedback from pilot runs. As one of a few studies [11], we explicitly assessed SAEs and USEs. We did not register any SAE, despite a few short-term USEs in the form of negative emotional reactions to a suicidal peer. Furthermore, the HEYLiFE program was conceived to be affordable and easily deliverable. HEYLiFE was also well-accepted among adolescents, thus substantially enhancing the options for effective prevention, at least in German-speaking countries.

This study has several limitations. The effects of the SARS-CoV2-pandemic on mental health may have confounded the outcomes of our evaluation. Previous findings suggested an increase in depression, anxiety, and PTSD among adolescents after pandemic onset [58]. Lower rates of inpatient care, but higher use of crisis services were reported in Germany [59]. Consistently, students in our sample who completed follow-up after the onset of the pandemic compared to before had higher levels of depression (PHQ-9) and risks factors for suicidality (RFS). Even if more students of the IG completed T2 after the onset of the pandemic compared to the CG, the IG did have a less steep increase in risk factors of suicidality than the CG from T0 to T2. This could indicate that the HEYLiFE prevention program promoted problem-solving and coping with difficulties, ultimately preventing students from reacting with an elevated level of suicidality to the pandemic. However, it is not possible clear how the pandemic affected mental health related outcomes and help-seeking in our sample. To reduce barriers to study participation, data collection on T1 took place only in the IG. 15.6% of the sample was aged >18 years. We decided to include all participants independently of age to keep a sample representative for the German school system and sufficient power in our analyses. Although the number of older adults was limited, it may have affected the results, since some of the outcomes vary by age groups [60, 61]. The analyses with gender and age-group as predictor were post-hoc and exploratory, so results should be interpreted with caution. The short-term effects are based on a pre-post comparison, and effects of repeated measurements cannot be ruled out. The psychometric properties of our German translation of the questionnaires could differ from the original instruments. Following the recommendations about appropriate cross-cultural adaptation and translation of questionnaires, the use of focus groups or more extensive back-translation procedures could have improved the quality of our translations of the stigma, knowledge, and attitudes questionnaire [62]. The internal consistency of the attitudes and stigma scores were rather low, which hampers the interpretability of the results. Our questionnaire on risk-factors showed promising psychometric properties in this study but depicted only a part of the mental states that contribute to suicidality. These observations on the psychometric properties of our questionnaires should be considered a source of potential bias. Moreover, sample selection may be biased since 42% of eligible students participated in the study, mainly due to lack of parental consent. This indicates a moderate level of acceptance for suicide prevention programs among parents, albeit concurrent data collection may pose a further obstacle to participant rates. As an alternative, passive parental consent forms should be explored, as well as the embedding of suicide prevention interventions in communal or national prevention strategies. Another selection bias occurred for T2 assessments, which were more likely to be completed when administered during school hours. Furthermore, the Covid-19-pandemic resulted in a significant dropout rate and a reduction in comparability between the IG and the CG. Besides, it caused a significantly longer time-interval between T0 and T2 in the IG (mean: 9 months ca.) than in the CG (mean: 6 months ca.). However, this does not undermine the interpretation of our findings: in fact, the significant effects observed after 9 months suggest that the HEYLiFE program can have an impact on outcomes across an even longer period.

## Conclusions

HEYLiFE is a promising intervention for suicide prevention in secondary schools. The level of acceptance in adolescents was high, and we found mid-term positive effects on suicide related variables, such as help-seeking intentions and risk-factors for suicidality. These results, along with the absence of SAEs, encourage the further development and dissemination of the HEYLiFE prevention program. Further development of the program should focus on minimizing unwanted short-term negative effects on stigma, targeting the needs of younger students and male students, and stabilizing the effects on knowledge and attitudes over time. The program was developed as a universal prevention approach. In the forthcoming evaluation phase evaluation, crucial steps will encompass systematically examining efficacy within subgroups with different levels of risk for suicidality or different age. Further perspectives include the combination of the program with an education program for teachers and social workers, or the analysis of health-economic benefits.

### Supplementary Information

**Additional file 1.** Items of the Risk Factor Scale (RFS).

**Additional file 2.** Unwanted Side-Effects and Serious Adverse Events.

**Additional file 3.** Short- (T1) and Mid-term Effects (T2) of the Intervention on SOSa items.

**Additional file 4.** Mean Difference between T0 and T2 for Both Groups for Outcomes with a Significant Time*Group Interaction.

**Additional file 5.** Fixed Effects of Group and Time on Lifetime Suicidality.

**Additional file 6.** Short- (T1) and Mid-term Effects (T2) of the Intervention with Gender as a Main and Interaction Term.

**Additional file 7.** Significant Interaction Effects of Gender on the Short-Term.

**Additional file 8.** Significant Interaction Effects on Gender on Mid-Term.

**Additional file 9.** Short- (T1) and Mid-term Effects (T2) of the Intervention with Age Group (12-13 y., 14-16 y., 17+ y.) as a Main and Interaction Term.

## Data Availability

The datasets generated and/or analysed during the current study are not publicly available due to institutional privacy requirements but are available from the corresponding author on reasonable request.

## Abbreviations

AHSQ: Actual Help Seeking Questionnaire
CG: control group
ERMIS: Emotional Reactions to the Mental Ill Scale
GHSQ: General Help Seeking Questionnaire
GLMM: generalized linear mixed models
IG: intervention group
LMM: linear mixed models
PHQ: Patient Health Questionnaire
PSS: Paykel’s Suicide Scale
PTSD: post traumatic stress disorder
RCT: randomized controlled trial
RFS: Risk Factors Scale
SAE: severe adverse events
SDQ: Strengths and Difficulties Questionnaire
SDS: Social Distance Scale
SOSa: Signs of Suicide questionnaire, attitudes scale
SOSk: Signs of Suicide questionnaire, knowledge scale
T0: baseline assessment
T1: post assessment (immediately after the intervention)
T2: 6-9 months follow-up assessment
USE: unwanted side effects
